# Protective Roles of Hydrogen Sulfide in Alzheimer’s Disease and Traumatic Brain Injury

**DOI:** 10.3390/antiox12051095

**Published:** 2023-05-13

**Authors:** Bindu D. Paul, Andrew A. Pieper

**Affiliations:** 1 Department of Pharmacology and Molecular Sciences, Johns Hopkins University School of Medicine, Baltimore, MD 21205, USA; 2 The Solomon H. Snyder Department of Neuroscience, Johns Hopkins University School of Medicine, Baltimore, MD 21205, USA; 3 Department of Psychiatry and Behavioral Sciences, Johns Hopkins University School of Medicine, Baltimore, MD 21205, USA; 4 Lieber Institute for Brain Development, Baltimore, MD 21205, USA; 5 Brain Health Medicines Center, Harrington Discovery Institute, University Hospitals Cleveland Medical Center, Cleveland, OH 44106, USA; andrew.pieper@harringtondiscovery.org; 6 Department of Psychiatry, Case Western Reserve University, Cleveland, OH 44106, USA; 7 Geriatric Psychiatry, GRECC, Louis Stokes Cleveland VA Medical Center, Cleveland, OH 44106, USA; 8 Institute for Transformative Molecular Medicine, School of Medicine, Case Western Reserve University, Cleveland, OH 44106, USA; 9 Department of Pathology, Case Western Reserve University, School of Medicine, Cleveland, OH 44106, USA; 10 Department of Neuroscience, Case Western Reserve University, School of Medicine, Cleveland, OH 44106, USA; 11 Translational Therapeutics Core, Cleveland Alzheimer’s Disease Research Center, Cleveland, OH 44106, USA

**Keywords:** transsulfuration, cysteine, hydrogen sulfide, Alzheimer’s disease, Huntington’s disease, traumatic brain injury, mitochondria, neuroprotection, learning and memory, dementia

## Abstract

The gaseous signaling molecule hydrogen sulfide (H_2_S) critically modulates a plethora of physiological processes across evolutionary boundaries. These include responses to stress and other neuromodulatory effects that are typically dysregulated in aging, disease, and injury. H_2_S has a particularly prominent role in modulating neuronal health and survival under both normal and pathologic conditions. Although toxic and even fatal at very high concentrations, emerging evidence has also revealed a pronounced neuroprotective role for lower doses of endogenously generated or exogenously administered H_2_S. Unlike traditional neurotransmitters, H_2_S is a gas and, therefore, is unable to be stored in vesicles for targeted delivery. Instead, it exerts its physiologic effects through the persulfidation/sulfhydration of target proteins on reactive cysteine residues. Here, we review the latest discoveries on the neuroprotective roles of H_2_S in Alzheimer’s disease (AD) and traumatic brain injury, which is one the greatest risk factors for AD.

## 1. Introduction

The mere mention of hydrogen sulfide (H_2_S) conjures up images of rotten eggs and sewers. H_2_S is a colorless, but not odorless, gas with the smell of rotten eggs and potent toxicity at high concentrations. In 1713, the Italian physician Bernardino Ramazzini first described H_2_S toxicity when he reported eye inflammation in workers who cleaned “privies and cesspits” (Ramazzini B. De Morbis Artificum Diatriba. Mutinae (Modena). Antonii Capponi, 1700 [1]). It was later discovered that H_2_S can be produced by both bacteria and eukaryotes and that the transsulfuration pathway mediates the interconversion of homocysteine and cysteine via the intermediate cystathionine [2,3]. This was followed by further biochemical characterization of the mammalian enzymes involved in H_2_S synthesis by several independent research groups [4,5,6,7]. 

Traditionally studied in readily accessible peripheral organs such as the liver, investigation of H_2_S in the brain gained momentum when Warenycia and colleagues observed that the inhalation of H_2_S by rats increased brain sulfides in direct proportion to the dose of gas inhaled and also mortality [8]. These investigators further revealed the presence of endogenous sulfides in normal brains without the inhalation of H_2_S, suggesting for the first time a normal physiologic role for H_2_S in the brain. Additionally, around that same time, elevated sulfide levels were observed in the brain of two fatal cases of H_2_S poisoning, further generating recognition and interest in the role of H_2_S in the brain [9]. Taken together, the detection of sulfides in the brain spurred detailed studies that ultimately forged a new field of research into this ancient signaling molecule [10]. 

Initial studies on H_2_S predominantly revolved around its toxic effects in mammals [11,12,13], most notably with respect to mitochondrial bioenergetics [14,15]. Indeed, at high concentrations, H_2_S inhibits cytochrome c oxidase and uncouples oxidative phosphorylation, which decreases adenosine triphosphate( ATP) production [16,17]. At lower concentrations, however, H_2_S exerts stimulatory and protective effects on mitochondria [18,19,20]. For example, H_2_S stimulates mitochondrial bioenergetics in multiple ways, including enhancing mitochondrial biogenesis and promoting mitophagy of damaged mitochondria [18]. Depending on the method and condition used, H_2_S levels can range anywhere from nanomolar levels to micromolar levels, with physiologic levels of H_2_S in the nanomolar range (reviewed in [21]). There is a dire need for specific and sensitive probes that can be utilized to detect and measure H_2_S in vivo. Diminished H_2_S production has been observed in several neurodegenerative diseases, including Huntington’s disease (HD), Parkinson’s disease (PD), and Alzheimer’s disease (AD). On the other hand, elevated H_2_S is also deleterious. For instance, the triplicated gene for cystathionine β-synthase (CBS) on chromosome 21 in Down syndrome increases H_2_S levels throughout the body, which suppresses complex IV of the mitochondrial electron transport chain [22,23,24]. This article focuses on the neuroprotective roles of H_2_S in vivo in the brain and its protective efficacy in both AD and traumatic brain injury (TBI), one of the leading risks factors for AD [25].

## 2. Biosynthesis of H_2_S in the Brain

**Figure 1:**
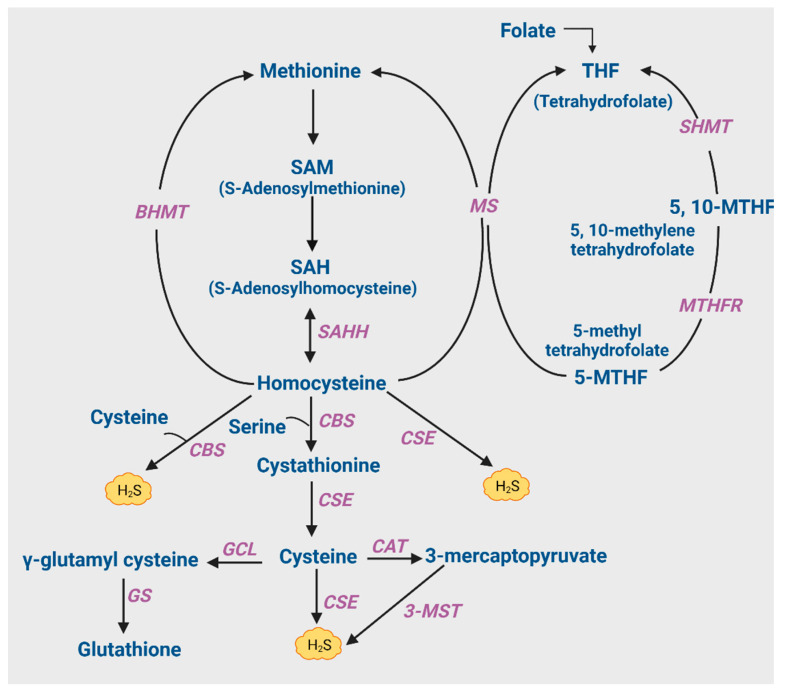
The transsulfuration and transmethylation pathways. Transsulfuration involves transfer of sulfur from homocysteine to cysteine. Homocysteine is generated from dietary methionine via S-adenosylmethionine (SAM) and S-adenosylhomocysteine (SAH). SAH is converted to homocysteine by S-adenosylhomocysteine hydrolase (SAHH). Homocysteine is then used either for generation of H_2_S via the transulfuration pathway or moved into the transmethylation pathway to generate methionine. With respect to the transulfuration pathway, cystathionine β-synthase (CBS) converts homocysteine to H_2_S or condenses homocysteine with serine to form cystathionine, which is then converted to cysteine by cystathionine γ-lyase (CSE). Cysteine is directly utilized by CSE to form H_2_S or alternatively converted to 3-mercaptopyruvate by cysteine aminotyransferase (CAT). 3-mercaptopyruvate sulfurtransferase (3-MST) then converts 3-MST to H_2_S. Cysteine can also be utilized to produce glutathione (GSH) by the sequential actions of glutamyl cysteine ligase (GCL) and GSH synthase (GS). The other pathway for homocysteine involves the regeneration of methionine via the transmethylation pathway. Methylation of homocysteine may occur either through a folate-independent or dependent pathway. In the folate-dependent pathway shown here, the vitamin B_12_-dependent enzyme methionine synthase (MS) converts homocysteine to methionine and tetrahydrofolate (THF), utilizing 5-methyltetrahydrofolate (5-MTHF) as the methyl donor. Next, serine hydroxymethyltransferase (SHMT) converts THF to 5,10-methylenetetrahydrofolate (5,10-MTHF), utilizing serine and vitamin B_6_. 5,10-MTHF is reduced to 5-MTHF by 5,10-methylenetetrahydrofolate reductase (MTHFR), remethylating another molecule of homocysteine in the process [29].

H_2_S is generated in vivo in the brain by three enzymes: cystathionine γ-lyase (CSE), CBS, and 3-mercaptopyruvate sulfur transferase (3-MST) through a coordinated and highly regulated process known as the transsulfuration pathway [7,26,27,28,29] (Figure 1). Notably, the expression of the biosynthetic enzymes for H_2_S is spatially compartmentalized. Specifically, CSE is exclusively localized to neurons, CBS is expressed in Bergmann glia and astrocytes [30,31], and 3-MST has also been localized to neurons [32]. All three enzymes utilize cysteine directly or indirectly to produce H_2_S. 3-MST acts on 3-mercaptopyruvate (3-MP) produced by cysteine aminotransferase (CAT) to produce H_2_S. In the absence of CAT, 3-MST cannot produce H_2_S unless 3-MP is present. Apart from these pathways, H_2_S can also be generated from the acid labile pool, iron–sulfur protein clusters, and the sulfane sulfur pool in the presence of endogenous reductants [33,34]. Higher levels of bound sulfur are found in the brain, with free H_2_S being maintained at low levels under basal conditions. H_2_S is also released from bound sulfur in homogenates of neurons and astrocytes under alkaline conditions, akin to what occurs in vivo when high extracellular concentrations of K^+^ ions are released by neuronal excitation [34]. The significance of the spatial compartmentalization of CSE, 3-MST, and CBS in the brain is not clear, and these enzymes may have cell-type-specific roles that are yet to be identified. It is increasingly evident that astrocytes have important signaling roles, ranging from immune signaling, synaptic plasticity, and metabolic functions to providing a support system for neurons [35,36,37]. The role of CBS in these processes is yet to be elucidated. Additionally, of the three H_2_S-generating enzymes, CSE is highly inducible, whereas CBS is constitutively expressed [27]. CBS can be regulated at the post-translational level by S-adenosyl methionine (SAM), an allosteric modulator [38]. Levels of SAM are decreased in the cerebrospinal fluid and brain tissue of AD patients, which could lead to a decrease in H_2_S levels [39,40]. 

## 3. H_2_S Signaling in Cognitive Functions

One of the earliest studies on the neuromodulator action of H_2_S in the central nervous system, conducted by Kimura and associates in 1996, showed that H_2_S acted on N-methyl D-aspartate (NMDA) receptors to modulate long-term potentiation and neurotransmission [10]. The same group also reported that H_2_S diminished oxidative stress in neurons [41], which was later attributed to the restoration of glutathione (GSH) levels and the stabilization of the mitochondria [42]. Oxidative stress plays a central role in pathogenesis of several neurodegenerative diseases, and thus restoring dysregulated H_2_S metabolism in these conditions was proposed to be beneficial [43]. However, it is important to note that H_2_S exhibits a bell-shaped dose response curve, with lower concentrations being generally beneficial and higher doses being toxic. The importance of this equilibrium is underscored by the tight regulation of endogenous H_2_S synthesis. Here, we review the current understanding of the role of H_2_S in two major forms of neuropsychiatric disease characterized by cognitive impairment: AD and TBI.

### 3.1. Alzheimer’s Disease

Alzheimer’s disease (AD), a multifactorial neurodegenerative condition characterized by the progressive loss of cognitive function and memory, is the leading worldwide cause of dementia [44,45]. Indeed, approximately 6.5 million Americans aged 65 and older are living with Alzheimer’s dementia today [46]. The neuropathological hallmarks of Alzheimer’s disease classically include the deposition of neurofibrillary tangles and paired helical fragments comprising the microtubule associated protein tau as well as the accumulation of plaques composed of Aβ peptides [47,48]. However, several lines of evidence also reveal that cognitively normal individuals often have deposits of amyloid plaques, and efforts to treat AD through addressing these two major forms of pathology have not yet succeeded [49,50]. The utility and safety of amyloid-based approaches has also recently been called into question by the Alves et al., whose meta-analysis of the published literature has shown that these therapeutic approaches may compromise long-term brain health by accelerating brain atrophy [51]. 

Notably, several studies have shown that the suboptimal or excessive levels of metabolites or cofactors of the transsulfuration pathway are also linked to dementia and cognitive deficits, pointing towards another possible therapeutic target for AD (Figure 1). For example, as described below, hyperhomocysteinemia has been linked to dementia.

#### 3.1.1. Hyperhomocysteinemia and Its Causes

Homocysteine is formed from S-adenosyl homocysteine by S-adenosylhomocysteine hydrolase (SAHH), and hyperhomocysteinemia has been established as an independent risk factor for dementia and cardiovascular diseases. Homocysteine is utilized as a substrate for both CSE and CBS to produce H_2_S. While CBS utilizes a combination of cysteine and homocysteine to produce H_2_S, CSE utilizes homocysteine alone to generate the gasotransmitter [52,53]. A subset of patients displaying homocysteinemia exhibited diminished CBS activity and substantial cardiovascular disability [54]. Thus, both elevated homocysteine levels and decreased H_2_S levels mediated vascular complications observed in subjects with impaired CBS activity [55]. Several studies have also shown that levels of homocysteine correlated with the severity of AD [56,57], and increased serum homocysteine was generally observed by the time people reached their nineties [58,59,60,61]. Elevated homocysteine levels additionally occur in TBI, one of the greatest risk factors for AD. Homocysteine can elicit toxicity in multiple ways, including the aberrant processing of the amyloid precursor protein (APP), overactivation of the NMDA receptor, DNA damage, and oxidative stress [29]. 

As described above, homocysteine resides at the intersection of the transsulfuration and transmethylation pathways, where it is either converted to cysteine via cystathionine or remethylated to methionine (Figure 1). Hyperhomocysteinemia may arise due to several reasons, including decreased expression or activity of the enzymes methylenetetrahydrofolate reductase (MTHFR), methionine synthase (MS), CBS, or CSE. MTHFR acts on 5,10-methylenetetrahydrofolate to produce 5-methyltetrahydrofolate, the methyl donor for remethylation of homocysteine to form methionine by MS. Notably, mutations in the *MTHFR* gene, which decrease the activity of the protein, have been linked to AD [62]. Along similar lines, mutations in the *CTH* gene are also associated with AD [63,64]. Decreased levels of folate, vitamin B_12_ (a cofactor for MS), or S-adenosyl methionine (SAM) also lead to hyperhomocysteinemia. SAM is a methyl donor generated from S-adenosylhomocysteine (SAH), which also serves as a cofactor for CBS activity [38]. SAM levels are decreased in AD, which may compromise the levels of cystathionine formed from homocysteine by CBS as well as H_2_S production because CBS utilizes homocysteine along with cysteine to produce H_2_S [39,40,53]. The administration of SAM to the APP/Presenilin-1 (PS1) mouse model of AD and to cultured astrocytes conferred cellular protection and stimulated the transsulfuration pathway [65]. Similarly, in both an Aβ intrahippocampal injection rat model and cultured SH-SY5Y cells, SAM enhanced GSH levels and prevented inflammatory changes and oxidative stress [66]. 

#### 3.1.2. Cysteine and GSH Metabolism

GSH, a tripeptide of glycine, glutamate, and cysteine, is one of the most abundant antioxidants in cells and tissues [67]. Among its many roles, GSH serves as a cofactor for several enzymes, modulates redox balance in cells, and provides a reservoir for glutamatergic neurotransmission [68]. The availability of cysteine is the rate limiting step for the synthesis of GSH, and, therefore, any alterations in cysteine may compromise GSH metabolism and elevate oxidative stress [69]. Aging, the biggest risk factor for developing AD, has also been associated with declining cysteine levels, and, as such, it has been designated as a “cysteine deficiency syndrome” [70]. Dysregulated cysteine metabolism has been observed in AD as well [71,72]. Cysteine levels are also regulated by the activity of its transporters, which exhibit suboptimal activity in several models of AD. Furthermore, soluble Aβ oligomers inhibit both basal and insulin-like growth factor-1 (IGF-1)-stimulated cysteine uptake through the neuronal cysteine transporter, the excitatory amino acid transporter 3 (EAAT3/EAAC1) [73]. Another study reported an increase in detergent-insoluble EAAC1 in the hippocampus of AD patients compared to normal subjects [74]. 

Along with declining cysteine levels, GSH is also significantly diminished in mouse models of AD prior to amyloid-β deposition, with the magnitude of the decrease in GSH correlating exponentially with the magnitude of increased intraneuronal Aβ accumulation [75]. Decreased GSH and antioxidant enzymes have also been linked to disease progression in AD [76]. While one meta-analysis of studies of postmortem tissues from subjects with mild cognitive impairment (MCI) and AD did not detect significant changes in GSH [77], it is important to note that the postmortem preservation of metabolites is notoriously variable. By contrast, recent magnetic resonance spectroscopy (MRS) studies have revealed significant depletion of brain GSH in MCI and AD, including the highly affected hippocampal brain region [78,79,80,81]. GSH depletion may also compromise the activity of GSH-requiring enzymes, such as glyoxalase 1 and 2 (GLO1 and 2), which are dysregulated in AD [82]. GLO1 and GLO2 are a part of the glyoxalase system that prevents glycation reactions mediated by alpha-ketoaldehydes such as methylglyoxal and glyoxal [83].

#### 3.1.3. H_2_S and AD

Although disrupted H_2_S balance in AD was reported several decades ago, the molecular mechanisms are only beginning to be understood [29]. Diminished brain H_2_S levels in AD were first observed in 2002 and correlated to decreased CBS activity [84]. Decreased H_2_S was also observed in the plasma of AD patients and correlated with severity of disease [85]. The same study also observed an increase in homocysteine levels. Diminished H_2_S that correlated with brain energy levels in the APP/PS1 mouse model of AD was additionally demonstrated using a fluorescent probe that simultaneously measured ATP [86]. In addition, decreased CSE levels have been observed in different models of AD, as described below [72,87].

Initial studies investigating the neuroprotective role of H_2_S in AD involved the administration of H_2_S donors, which exerted protective effects in several AD models. In a rat model involving the hippocampal injection of Aβ1-40, for example, the fast release H_2_S donor sodium hydrosulfide (NaHS) prevented neuroinflammation by suppressing the increase in cytokines, such as Tumor necrosis factor alpha (TNF-α), interleukin 1-beta (IL-1β), IL-6, and pro-inflammatory cyclooxygenase-2 (COX-2) [88]. NaHS also prevented amyloid-β-induced toxicity in microglial cultures, reduced inflammation, preserved mitochondrial function [89], and prevented Aβ-induced toxicity in PC12 cells [90,91]. Furthermore, NaHS prevented spatial memory impairment and neuroinflammation in an amyloid-based rat model of AD [92]. A separate study utilizing NaHS and Tabiano’s spa-water (rich in sulfur compounds) ameliorated behavioral deficits in three experimental models of AD [93]. H_2_S donors were also tested in a *C. elegans* model of AD and shown to reduce Aβ aggregation, increase levels of acetylcholine, and reduce oxidative stress [94]. Another study reported that dietary methionine restriction in APP/PS1 mice ameliorated neurodegeneration, improved cognition, and increased H_2_S production [95]. Several studies have also reported on the beneficial effects of natural products or plant-based foods that are rich sources of H_2_S, including garlic [96]. In 2007, for example, it was demonstrated that the garlic-derived compounds diallyl sulfide (DAS), diallyl disulfide (DADS), and diallyl trisulfide (DATS) all release H_2_S, with diallyl trisulfide (DATS) showing the greatest activity [97,98]. S-allylcysteine (SAC), another garlic compound, also increases H_2_S by increasing expression of CSE [99,100]. After these findings, garlic extracts and their endogenous sulfur containing compounds were tested and found to be efficacious in models of AD [101,102,103,104,105,106]. A summary of beneficial effects of H_2_S in models of AD is shown below in Table 1.

##### Persulfidation, AD, and Aging

One of the modes by which H_2_S signals is through post-translational modification, termed sulfhydration or persulfidation, which both refer to the same chemical reaction in which the thiol group of cysteine is converted to an –SSH or persulfide group [52,120,121]. Here, H_2_S cannot directly modify an –SH group, and the main mechanisms by which persulfides are generated are by reactions of H_2_S with oxidized cysteines or disulfides or by reactions of cysteine residues with sulfide radicals, polysulfides, and other persulfides [121,122]. Persulfidation is a reversible modification regulated by the endogenous thioredoxin/thioredoxin reductase system [123,124,125]. Notably, persulfidation and H_2_S signaling are compromised in several age-related neurodegenerative diseases, including AD, HD, PD, and spinocerebellar ataxia (SCA) [126,127,128,129]. 

**Figure 2:**
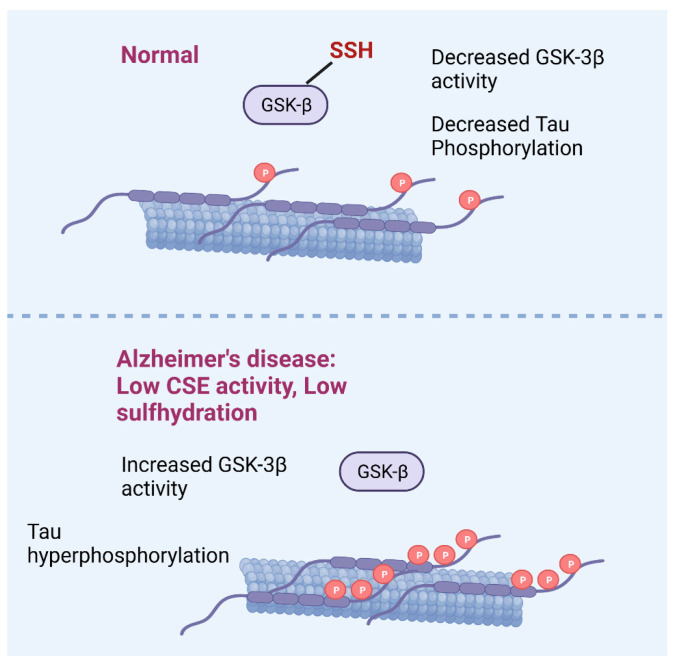
Disruption of H_2_S signaling in Alzheimer’s disease (AD). During normal conditions, glycogen synthase kinase-3β (GSK-3β) is sulfhydrated, which inhibits its catalytic activity and prevents hyper-phosphorylation of Tau (Top panel). In AD, decreased CSE activity leads to lower sulfhydration of GSK-3β, which leads to elevated phosphorylation of Tau.

Although there are numerous studies on the beneficial effects of H_2_S in AD, analysis of persulfidation (the major mode by which the gaseous molecule signals) in AD is scarce. More recently, the status of persulfidation has been studied directly in AD (Figure 2). For example, overall persulfidation is decreased in mouse models of AD as well as postmortem samples from human AD subjects [72]. Notably, persulfidation of Glycogen synthase kinase-3β (GSK-3β), the major kinase that phosphorylates Tau, is decreased in AD. Administration of Na-GYY4137 to the 3xTg-AD model of AD rectified the persulfidation deficits and prevented motor and cognitive decline [29,72]. Suboptimal persulfidation was also observed in the APP/PS1 mouse model of AD, where decreased expression of CSE was observed, in addition to the human hippocampal and cortex samples [87]. This study also demonstrated a role for the autophagy-related activating factor 6 (ATF6) in expression of CSE. Stereotaxic injection of lentiviral particles encoding CTH into the ventricular system of the brain rescued spatial memory deficits in *Atf6* CKO/APP/PS1 mice. The transport of ATF6 from the endoplasmic reticulum to the Golgi apparatus under ER stress is mediated by the Soluble NSF attachment protein (αSNAP)/ SNAP receptor (SNARE) pathway. Persulfidation of αSNAP is also decreased in ATF6 knockout mice, which may indicate a role in AD. 

Lastly, aging is the biggest risk factor for developing AD, and diminished persulfidation is also ubiquitously observed across evolutionary boundaries during aging. Importantly, several of the proteins whose persulfidation levels are altered may modulate AD pathology and disease progression [122,130]. A systematic analysis of these targets is warranted to elucidate the links between persulfidation and AD. For instance, MTHFR, a key enzyme regulating intracellular homocysteine metabolism, is normally persulfidated, and decreased persulfidation of MTHFR promotes hyperhomocysteinemia, a risk factor for AD [131]. 

### 3.2. Traumatic Brain Injury

#### TBI and H_2_S

Traumatic brain injury (TBI) is one of the most prevalent forms of neurodegenerative disease, and typically entails chronic and progressive neuropsychiatric impairment even after a single injury. This pathologic process is poorly understood, and, to date, there are no protective treatments for patients. It is well-established that there is considerable overlap in pathology between TBI and AD [25], and H_2_S is involved in various biological functions after TBI, including the response to oxidative stress in the brain. Due to the brain’s voracious need for the constant generation of ATP, which generates free radicals as a by-product, and the brain’s abundance of metal ions and phospholipids that generate additional oxidative products, the brain, even under normal circumstances, is constantly exposed to high levels of oxidative stress. This vulnerability is further exacerbated by TBI because of an increased energy requirement for self-repair processes after injury that ultimately leads to an inability to maintain mitochondrial membrane potential, resulting in complete energy failure and cell death. Early results in cellular models indicated protective efficacy of H_2_S in this scenario. For example, H_2_S generated by mitochondrial 3-MST directly reduced the generation of ROS and protected PC12 cells from apoptosis after severe oxidative stress [132]. In cultured neurons, H_2_S promoted the neuronal production of GSH and the scavenging of oxygen free radicals, hydrogen peroxide, and lipid peroxides [42,133]. Thus, whether changes in brain H_2_S might be involved in the pathophysiology of TBI, and whether supplemental H_2_S might be neuroprotective in TBI, became an active area of investigation.

There is now substantial evidence in animal models that TBI decreases brain levels of H_2_S and that exogenous supplementation of H_2_S protects the brain after injury. In 2013, Zhang et al. observed that subjecting mice to the weight drop model of TBI acutely decreased levels of hippocampal and cortical CBS mRNA and protein, and this correlated with decreased levels of H_2_S [134]. They additionally demonstrated that pretreatment with the H_2_S donor NaHS partially reduced lesion volume after injury [134]. That same year, Jiang et al. showed the protective efficacy of NaHS in an additional model of TBI induced by controlled cortical impact (CCI) in rats [135]. Specifically, CCI acutely decreased brain H_2_S, and the preservation of brain H_2_S with NaHS treatment beginning before injury blocked brain edema, blood–brain barrier (BBB) impairment, and the acquisition of motor deficits. They also reported that NaHS-treated rats showed reduced TBI lesion volume and were protected from TBI-induced decreases in brain superoxide dismutase and choline acetyltransferase activity, as well as TBI-induced increases in the oxidative products 8-iso-prostaglandin F2 alpha and malondialdehyde, up to 72 h after injury [135]. 

**Figure 3:**
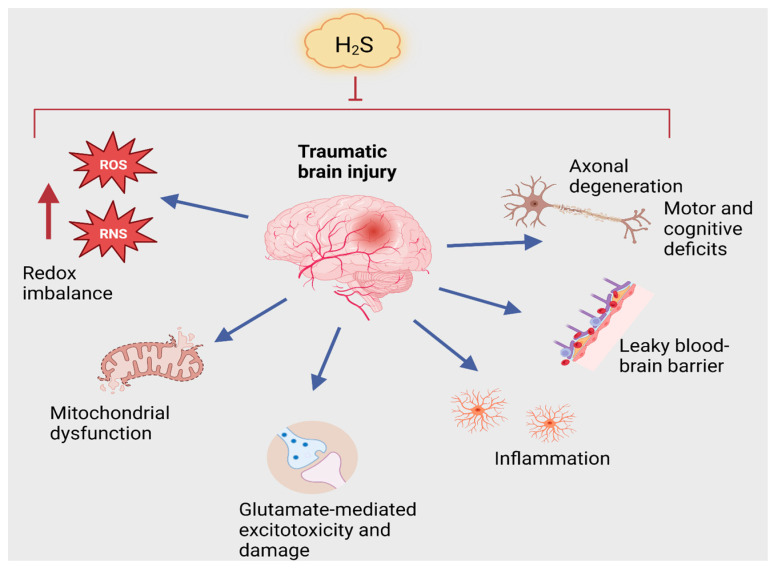
Neuroprotective effects of H_2_S in traumatic brain injury (TBI). TBI impacts several physiological processes that lead to motor and cognitive deficits. TBI causes increases in reactive oxygen and nitrogen species (ROS and RNS), leading to oxidative and nitrosative stress and mitochondrial dysfunction. TBI also elicits glutamate-induced neurotoxicity and inflammation and causes a leaky blood–brain barrier. All of these processes influence each other and ultimately culminate in neurodegeneration.

These results were further bolstered when Zhang et al. extended their work in the weight drop model of TBI the following year, showing that NaHS pretreatment prevented cerebral edema and cognitive impairment in the Morris water maze after TBI, as well as cleavage of caspase-3, decreased Bcl-2, and elevated neuronal apoptosis [136]. Preserved cognition in the Morris water maze by NaHS pretreatment in rats exposed to CCI was also independently established by the Hajisoltani laboratory [137], and Xu et al. additionally demonstrated that NaHS-mediated modulation of the PI3K/Akt/mTOR signaling pathway after TBI in mice was associated with BBB protection, the inhibition of neuronal apoptosis, remyelination of axons, and preservation of mitochondrial function [138]. Evidence for protective efficacy of H_2_S in the CCI model of TBI was also demonstrated by Campolo et al., who showed that the administration of 2-(6-methoxynapthalen-2-yl)-propionic acid 4-thiocarbamoyl-phenyl ester (ATB-346), a H_2_S-releasing derivative of naproxen, attenuated TBI-induced brain edema, neuronal cell death, and motor impairment, while naproxen (which does not release H_2_S) had no protective efficacy [139]. ATB-346 was also associated with significantly decreased expression of inducible nitric oxide synthase (iNOS), cyclooxygenase 2 (COX-2), tumor necrosis factor alpha (TNFα), and interleukin 1-beta (IL-1β), as well as normalized levels of glial-derived neurotrophic factor (GDNF) and nerve growth factor (NGF), and increased levels of vascular endothelial growth factor (VEGF), after TBI [139]. While all of these changes would be considered likely beneficial to brain health in the setting of TBI, it is unclear whether any of these occur as a direct result of H_2_S action or if this simply reflects the profile of a healthier brain by virtue of other upstream effects of H_2_S. Interestingly, Zhang et al. have shown that increased expression of 3-MST in neurons occurs predominantly in those neurons that survive after TBI [140], implicating a regulated and direct protective role of endogenous H_2_S after TBI. More recently, H_2_S-mediated protection in TBI in rats has been linked to the modulation of glutamate-mediated oxidative stress via the p53/glutaminase2 pathway [141]. In addition, the Centurion laboratory recently reported that subchronic treatment with NaHS protected rats from hemodynamic and sympathetic nervous system impairments after TBI and also restored CSE and CBS expression in the brain [142]. It has also been demonstrated that subchronic NaHS after TBI in rats prevents hypertension, vascular impairment, and oxidative stress [143]. In conclusion, there are a myriad of central and peripheral beneficial effects of H_2_S on TBI outcomes, although the precise mechanisms by which these protective effects occur are currently unknown (Figure 3).

## 4. Dysregulation of Iron Homeostasis in AD, TBI and Intersection with H_2_S Signaling

Iron is a transition metal with important roles in the brain, ranging from being a component of iron–sulfur cluster proteins and heme proteins to participating in DNA synthesis and neurotransmitter metabolism. However, the dysregulation of iron homeostasis can be deleterious, as ferrous iron (Fe^2+^) reacts with H_2_O_2_ and produces ^•^OH and HO_2_ to oxidize lipids, proteins, and DNA [43]. Additionally, superoxide radicals (O_2_^•−^) produced by mitochondria during respiration reduce Fe^3+^ to Fe^2+^ by the Haber–Weiss reaction [144]. The accumulation of iron in the brain is a common feature of aging, several neurodegenerative diseases, and TBI and is known to drive neuronal loss [145,146,147,148,149]. Specifically, disrupted iron metabolism and its aberrant redox cycling trigger ferroptosis, an iron-dependent cell death pathway that elicits lipid peroxidation and damage to cellular components in several neurodegenerative diseases, including AD [150,151,152,153]. Ferroptosis was described over a decade ago as a distinct form of cell death linked to aging, neurodegeneration, immune system dysfunction, and cancer [151,154]. It is well-established that ferroptosis is intimately linked to depletion of cysteine, a component of GSH in cells, and several studies have shown that cysteine/cystine deprivation can elicit this form of cell death. As cysteine serves as the substrate for generation of H_2_S, the involvement of this gasotransmitter in ferroptosis has been explored. To date, H_2_S donors have been shown to alleviate damage caused by ferroptosis in various contexts by activating cytoprotective signaling pathways [155,156,157]. The effect of H_2_S on ferroptosis in the brain in the context of injury and neurodegeneration is yet to be systematically studied and could inform the development of novel therapeutics.

## 5. Therapeutic Opportunities

Although significant advances in the elucidation of signaling mediated by H_2_S have been made, clinical translation has yet to follow. While there is abundant evidence of the neuroprotective efficacy of H_2_S donors in rodents, *Drosophila,* and worm models, examples of translation to human disease are scarce. However, some H_2_S-donating hybrid drugs have made it into clinical trials, including a phase 2B study that demonstrated a reduction in gastrointestinal toxicity of the hybrid H_2_S-releasing analgesic/anti-inflammatory drug ATB-346, as compared to the non-steroidal anti-inflammatory drug (NSAID) naproxen that produces a similar inhibition of the inflammatory cyclooxygenase-2 (COX2) molecule. [158]. The safety and side-effects of these compounds are still being evaluated. Harnessing H_2_S donors can prove challenging, as the timing and dose of the donors likely requires optimization. For example, numerous reports have demonstrated a biphasic dose–response curve for H_2_S, with higher doses being toxic. An alternate approach involves the use of natural H_2_S donors such as garlic extracts, which are rich in sulfur compounds that release H_2_S and may be beneficial in cardiovascular disorders [97,98]. The use of such donors might also be considered in ameliorating symptoms of AD, TBI, and other neurodegenerative disorders involving diminished H_2_S signaling. The opposite may be true of diseases involving elevated H_2_S, such as Down syndrome, in which the trisomy of chromosome 21 leads to excess H_2_S production due to an extra copy of CBS [23,24]. Thus, depending on the paucity or excess of H_2_S, appropriate treatment strategies will need to be developed. 

## 6. Conclusions

AD is a complex, multifactorial disease, with most cases arising sporadically. The susceptibility factors for developing AD are several, with aging and TBI being major risk factors. There are several commonalities between AD and TBI, including dysregulated gasotransmitter signaling. Accumulating evidence shows that deficiencies in the gaseous signaling molecule H_2_S can drive pathology in AD and TBI and that the augmentation of H_2_S levels affords therapeutic benefits in these conditions. Stimulating H_2_S production or restoring the homeostasis of the various metabolites of the transsulfuration and transmethylation pathway that contribute to cysteine, GSH, or H_2_S production may be beneficial for these or other related forms of neurodegenerative disease. H_2_S is a gaseous molecule and cannot be stored in vesicles, unlike conventional neurotransmitters, but elicits effects through sulfhydration, which can be used as a marker for its action. Accurate measurement of the various forms and metabolites H_2_S would further deepen our knowledge pertaining to the physiological relevance of this gaseous messenger molecule.

## Figures and Tables

**Table 1:** Neuroprotective effects of hydrogen sulfide.

H_2_S Donor/ Boosting Agent	System	Effect	Ref.
NaHS	β (Aβ 25-35)-treated PC12 cells	Prevented β (Aβ 25-35)-induced cytotoxicity and apoptosis by reducing the loss of MMP and attenuating the increase in intracellular ROS.	[90]
NaHS	PC12 cells	Decreased beta secretase-1 (BACE-1) levels and Aβ1-42 release. Increased phosphorylation of Akt.	[91]
NaHS	Aβ1-40-induced toxicity in BV-2 microglial cells	Attenuated Aβ1-40-induced lactate dehydrogenase (LDH) release and expression of growth arrest DNA damage (GADD 153). Inhibited release of nitric oxide, induction of inducible nitric oxide synthase (iNOS), and expression of tumor necrosis factor α (TNF-α) and cyclooxygenase 2 (COX-2).	[89]
NaHS	Rat Amyloid β (Aβ 1-40)-injection model	Ameliorated learning and memory deficits. Suppressed Aβ(1-40)-induced apoptosis in the hippocampal CA1 region. Diminished hippocampal inflammation, astrogliosis, and microgliosis.	[92]
NaHS	APP/PS1 mouse model of AD	Improved spatial memory. Decreased expression of BACE1, PS1, and amyloidogenic C99 fragment. Increased expression of ADAM17 and nonamyloidogenic C83 fragment.	[107]
NaHS	APP/PS1 mouse model of AD	Increased hippocampal H_2_S levels, intracellular ATP, and mitochondrial COX IV activity. Improved hippocampus-dependent contextual fear memory and novel object recognition. Decreased Aβ accumulation in mitochondria.	[108]
NaHS	APP/PS1 mouse model of AD	Inhibited γ-secretase activity and decreases mitochondrial Aβ production in neurons.	[109]
NaHS	Rat amyloid β (Aβ 25-35)-injection model	Prevented neuronal loss, apoptosis, and activation of pro-caspase-3. Decreased expression of phosphodiesterase 5 (PDE-5) in the hippocampus. Upregulated expression of peroxisome proliferator-activated receptor (PPAR)-α and PPAR-γ. Prevented Aβ 25-35)-decrease in IκB-α degradation and increase in nuclear factor-κB (NF-κB) p65 phosphorylation levels.	[110]
NaHS	Primary hippocampal neuronal culture from C57/BL6 mice at P0-P1	Reduced Aβ-induced apoptosis. Decreased release of cytochrome C into the cytosol and caspase-3 activity. Decreased translocation of the phosphatase and tensin homologs deleted on chromosome 10 (PTEN) from the cytosol to the mitochondria. Increased p-AKT/AKT levels in PI3K-dependent manner.	[111]
NaHS	APP/PS1 mouse model of AD	Mitigated cognitive deficits. Decreased the number of senile plaques, Aβ1-40 and Aβ1-42 levels, neuronal loss, beta-amyloid precursor (APP), and BACE1. Increased CBS and 3MST. Augmented antioxidant effects through induction of nuclear factor erythroid-2-related factor 2 (Nrf2), heme oxygenase-1(HO-1), and glutathione S-transferase (GST).	[112]
Memit (Derived from memantine by replacing the free amine group with an isothiocyanate moiety)	Aβ oligomers-induced damage in human neurons and rat	Decreased Aβ(1-42)-induced aggregation and Aβ oligomer-induced damage in human neurons and rat microglial cells. Increased neuroprotective autophagy.	[113]
H_2_S releasing peptide conjugates	*C. elegans*	Reduced Aβ1–42 amyloid deposits and ROS. Increased acetylcholine levels.	[94]
Sulfanagen	APP/PS1 mouse model of AD	Stimulated 3-MST and prevented neuropathology.	[114]
Methionine restriction	APP/PS1 mouse model of AD	Decreased Aβ accumulation. Improved cognitive function. Restored synapse ultrastructure. Alleviated mitochondrial dysfunction by enhancing mitochondrial biogenesis in male mice. Balanced the redox status and activated cystathionine-β-synthase (CBS)/ H_2_S pathway.	[95]
Naringin	Rat Amyloid β (Aβ)-injection model	Improved spatial memory. Increased H_2_S production.	[115]
NaHS	LPS-induced AD-like cognitive deficits in Wistar albino rats	Reduced inflammation. Decreased oxidative stress, apoptosis, and histopathological alterations.	[116]
NaHS in combination with the NMDA-receptor antagonist, MK801	Homocysteine (intracerebral injection)-induced AD-like neurodegeneration	Ameliorated BBB disruption, impaired cerebral blood flow (CBF), and diminished synaptic function.	[117]
NaHS	Mouse primary hippocampal neurons (E16–18), 3xTg-AD mouse model of AD	Reduced neuronal death in Aβ1-42 treated primary hippocampal neuronal culture and increased neurite length. Increased plasma H_2_S and decreased anxiety-like behavior and cognitive deficits in 3xTg-AD mice. Reduced amyloid deposits and hyperphosphorylation of Tau. Decreased inflammatory responses, oxidative stress, and gliosis in 3×Tg-AD mice.	[118]
NaHS, sulfurous water	3xTg-AD mouse model of AD	Prevented learning and memory deficits. Reduced size of amyloid β plaques. Decreased activity of c-jun N-terminal kinases, extracellular signal-regulated kinases, and p38.	[119]
NaHS, Tabiano’s spa-water	Rat Aβ1–40 injection model of AD, Streptozotocin-induced AD, 3xTg-AD mouse model of AD	Improved learning and memory deficits in all three models. Decreased amyloid deposits in the rat models of AD. The spa-water decreased oxidative and nitrosative stress, MAPK activation, inflammation, and apoptosis in 3xTg-AD mice.	[93]
Na-GYY4137	3xTg-AD mouse model of AD	Improved motor and cognitive function in 3xTg-AD mice. Increased overall sulfhydration. Inhibited Tau phosphorylation by sulfhydrating glycogen synthase kinase (GSK3β) and inhibiting its activity.	[72]

## Abbreviations

3-MST	3-mercaptopyruvate sulfurtransferase
AD	Alzheimer’s disease
APP	Amyloid precursor protein
ATB346	2-(6-methoxynapthalen-2-yl)-propionic acid 4-thiocarbamoyl-phenyl ester
ATF6	Activating factor 6
BBB	Blood–brain barrier
CAT	Cysteine aminotransferase
CBF	Cerebral blood flow
CBS	Cystathionine β-synthase
CSE	Cystathionine γ-lyase
CCI	Controlled cortical impact
COX-2	Cyclo-oxygenase-2
DADS	Diallyl disulfide
DATS	Diallyl trisulfide
EAAT3/EAAC1	Excitatory amino acid transporter 3
GDNF	Glial-derived neurotrophic factor
GLO-1,2	Glyoxalase 1,2
GSH	Glutathione
GSK-3β	Glycogen synthase kinase-3β
HD	Huntington’s disease
iNOS	Inducible nitric oxide synthase
MCI	Mild cognitive impairment
MRS	Magnetic resonance spectroscopy
MTHFR	Methylenetetrahydrofolate reductase
NGF	Nerve growth factor
Nrf2	Nuclear factor erythroid-2-related factor 2
PD	Parkinson’s disease
PDE5	Phosphodiesterase 5
PS1	Presenilin-1
ROS	Reactive Oxygen Species
RNS	Reactive Nitrogen Species
SAC	S-allyl cysteine
SAHH	S-adenosylhomocysteine hydrolase
SAM	S-adenosyl methionine
SCA	Spinocerebellar ataxia
α-SNAP	α-soluble NSF attachment protein
SNARE	Soluble NSF attachment protein receptor
TNF-α	Tumor necrosis factor alpha
VEGF	Vascular endothelial growth factor
